# Radiofrequency Ablation Versus Partial Nephrectomy in Treating Small Renal Tumors: A Systematic Review and Meta-Analysis

**DOI:** 10.1097/MD.0000000000002255

**Published:** 2015-12-18

**Authors:** Xiaotao Yin, Liang Cui, Fanglong Li, Siyong Qi, Zhaoyang Yin, Jiangping Gao

**Affiliations:** From the Department of Urology, Chinese PLA General Hospital (XY, FL, SQ, ZY); Department of Urology, General Hospital of Civil Aviation of China (LC); and Department of Urology, The First Affiliated Hospital of PLA General Hospital, Beijing, China (JG).

## Abstract

Radiofrequency ablation (RFA) has emerged as an alternative treatment to surgical partial nephrectomy (PN) in the treatment of small renal tumors (SRTs). But its safety and oncological efficacy are still controversial.

We conducted this systematic review and meta-analysis to compare the peritoperative and oncological outcomes of RFA and PN in the treatment of SRTs.

Pubmed, EMBASE, Cochrane CENTRAL, and Web of Science were searched to identify eligible studies that compared the RFA and PN in the treatment of SRTs.

Twelve retrospective studies that compared RFA with PN in the treatment of SRTs met our selection criterion and were included in this meta-analysis. The pooled results indicated that the local recurrence rate (4.14% vs 4.10%, RR: 1.18, 95% CI: 0.68, 2.07, *P* = 0.550) and distant metastases rate (2.76% vs 1.89%, RR: 1.31, 95% CI: 0.70, 2.46, *P* = 0.686) were not significantly different between the RFA group and the PN group. In terms of perioperative outcomes, RFA was associated with shorter length of stay (LOS) (WMD: −2.02 days, 95% CI: −2.77, −1.27, *P* < 0.001), lower eGFR decline after treatment (WMD: −3.90, 95% CI: −6.660, −1.140, *P* = 0.006). However, the overall perioperative complication rate (7.5% vs 6.2%, RR:1.10, 95% CI: 0.64, 1.87, *P* = 0.740) and the major complication rate (3.7% vs 4.4%, RR: 0.83, 95% CI: 0.43, 1.60, *P* = 0.579) were both similar between RFA and PN groups.

Compared with PN, RFA achieves an equal oncological outcome for SRTs with similar local recurrence rate and distant metastases rate. Additionally, RFA is associated with a similar perioperative complication rate, lower decline of eGFR, and shorter LOS. Therefore, RFA is an effective option in the treatment of SRTs for selected patients.

## INTRODUCTION

With the development and the increased utilization of imaging modalities, such as ultrasonograghy (US), computed tomography (CT), and magnetic resonance imaging (MRI), the incidence of small renal tumors (SRTs), which refer to T1 stage renal tumors, has steadily increased in the past decades.^1,2^ And renal cell carcinoma (RCC), which is the third most common malignancy in the urogenital system, accounts for ∼70% of the SRTs.^3^

The management of RCCs has changed in the past several years. Nephron–sparing surgery (NSS) or partial nephrectomy (PN) has become the standard surgical approach for the localized RCCs, which can achieve the similar oncological control effect to radical nephrectomy (RN).^4,5^ Additionally, through the long-time follow-up of the patients receiving PN or RN, it was found that PN could independently decrease the risk of cardiovascular events relative to RN.^6^ Therefore, PN is recommended for the treatment of SRTs in guidelines of European Association of Urology (EAU).^7^ However, surgical PN is inevitably associated with some limitations. Perioperative complications occur in ∼20% of PN surgical cases, which result in significant mortality and high cost of health care system.^8,9^ Additionally, the warm ischemia of kidney may cause potential damage to the renal function. So, surgical PN is not suitable for some elderly and high-risk patients that have severe comorbidities and cannot tolerate anesthesia and surgical trauma.

Radiofrequency ablation (RFA), which destroys the tumor cells by heats, is increasingly used in the treatment of SRTs.^10^ RFA could be performed via percutaneous or laparoscopic routes, which avoid the incision of renal parenchyma and the clamp of renal vessels. So it is presumed that patients can recover more quickly after RFA with less complications. Therefore, RFA may be an effective option in the treatment of SRTs. However, there are still no randomized controlled trials of high quality comparing the safety and oncological efficacy between RFA and PN until now. It is still debated whether RFA can achieve equivalent safety and long-term tumor control efficacy. Recently Wang et al^11^ conducted a meta-analysis comparing oncologic outcomes and complications between RFA and PN. But the included studies in this meta-analysis were all single-arm observational studies, which introduced several biases and hampered the quality of the conclusion inevitably.

To obtain a more reliable comparison on the safety and oncological efficacy of RFA versus PN in the treatment of STRs, we conducted this systematic review and meta-analysis of published articles that directly compared RFA with PN in the treatment of SRTs.

## METHODS

### Search Strategy

This meta-analysis was complied with the guideline of Preferred Reporting Items for Systematic Reviews and Meta-Analyses (PRISMA).^12^ Because the data included in our study were extracted from published literatures, ethical approval from ethics committees was not needed.

A literature search for published original articles was conducted in Pubmed, EMBASE, Cochrane CENTRAL, and Web of Science. The last updated search was carried out on June 30, 2015. The following combinded search items through MeSH headings, keywords, and text words were used: (“radiofrequency” OR “RFA” OR “radio frequency” OR “thermal ablation”) AND (“nephrectomy” OR “nephron-sparing surgery” OR “NSS” OR “partial nephrectomy” OR “PN” OR “LRP” OR “RPN”). Additionally, references of relevant literatures were manually screened for further publications.

### Selection Criteria

The identified studies were included if they met the following criteria: (1) only cohort studies were considered for inclusion; (2) studies must compare the RFA with PN directly for the treatment of localized SRTs; (3) RFA could be performed via percutaneously or laparoscopic approach; PN consisted of open, laparoscopic, and robotic-assisted approaches; (4) studies must have a median follow-up time of more than 12 months; (5) each study must report the incidence of local recurrence and distant metastases, or the perioperative outcomes. The articles that were not written in English were excluded. Letters, case reports, meeting abstracts or review articles were also excluded. When duplicate articles were encountered, we included the more informative and latest article. Two researchers (Xiaotao Yin and Fanglong Li) screened titles and abstracts of all searched studies and identified the studies that met the selection criteria for next analysis. Discrepancies were resolved through discussion with Liang Cui.

### Quality Assessment

According to the Cochrane Handbook of nonrandomized studies, methodological quality of all the included studies was assessed independently by 2 researchers (Fanglong Li and Xiaotao Yin) using the Newcastle Ottawa Scale (NOS).^13^ The checklist point compromised the following 3 aspects: selection (up to 4 points), comparability (up to 2 points), and outcomes (up to 3 points). The maximum score of each study by NOS was 9 points, and the score of >5 points was considered to be adequately qualified for the meta-analysis. During the process discrepancies were resolved though discussion with Jiangping Gao.

### Data Extraction

The relevant information was extracted independently by 2 researchers (Siyong Qi and Zhaoyang Yin) using a predefined form. The information contained the first author's last name, year of publication, study location, type of study design, patient characteristics, length of stay (LOS), perioperative complications, histological results of tumors, decline of estimated glomerular filtration rate (eGFR), local recurrence, and distant metastases. Patient characteristics included age, gender distribution, number of patients in each group, tumor size, and follow-up. Discrepancies during data extraction were resolved by discussion through full discussion.

### Statistical Analysis

The statistical analysis was carried out by the use of STATA software, version12.0 (State Corporation, College Station, TX). For categorical variables, the count number or proportion were extracted from each study and merged in STATA. When the counts of zero were encountered, a fixed continuity correction with 0.5 was performed. And studies with zero total event were also included in the analysis to provide a more conservative estimate of effect size.^14^ For continuous variables, means and standard deviations (SD) were extracted from each study and merged. If the SD were not provided while the mean was available, the values of SD were estimated from the relevant data.

A test of heterogeneity of included studies was conducted using the Mantel–Haenszel chi square test and the Higgins *I*-*squared* statistics. *I*^*2*^ values >50% in Higgins *I*-squared statistics indicated the presence of significant heterogeneity among studies, and the random-effects model was applied for the meta-analysis.^15^ Otherwise, the fixed-effects model was used. The risk ratio (RR) was used to assess categorical data. For the comparison regarding continuous data, inverse variance models were used to obtain the estimated weighted mean difference (WMD). We also performed sensitivity analysis by sequential omission of individual study to evaluate the stability of the pooled results. Publication bias was also detected by visual inspection of funnel plots, Begg–Mazumdar adjusted rank correlation test,^16^ and Egger regression asymmetry test.^17^ For all analysis, *P* values < 0.05 was considered statistically significant.

## RESULTS

### The Search Results and Quality Assessment

**FIGURE 1:**
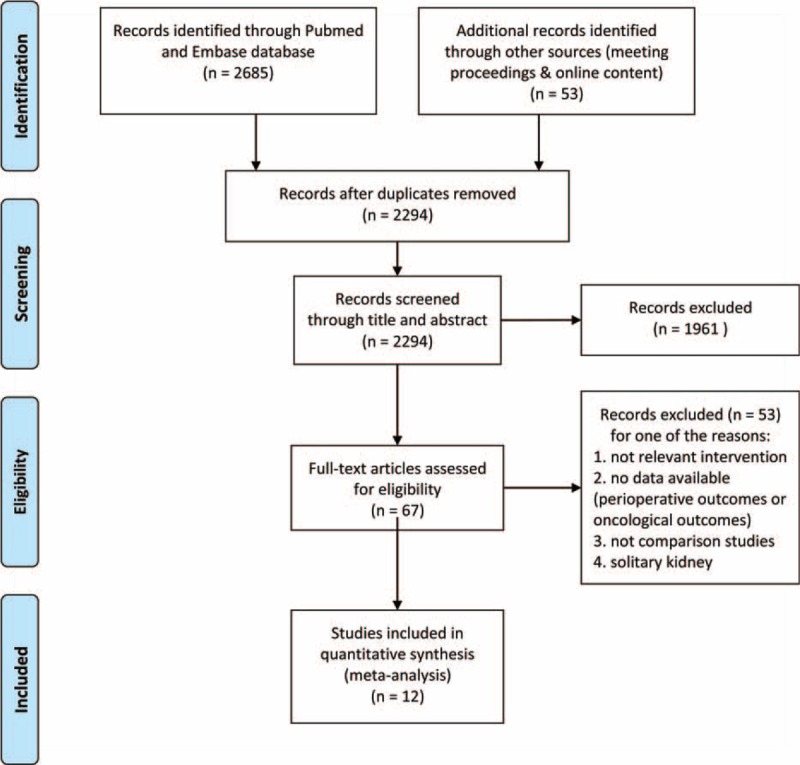
Flowchart of literature search and selection.

A total of 2738 records were identified after the primary comprehensive literature research using aforementioned strategy. A total of 444 duplicated items were excluded. After screening the titles and abstracts of identified records, 1961 studies were excluded for the reasons including animal studies, reviews, letters, case reports, conference abstracts, and other irrelevant studies. After full text assessment, 2 studies^18,19^ whose participants suffered from solitary kidney were excluded, and 12 studies^8,20–30^ which met our selection criterion were eventually included in our research. The flowchart of the study selection process was shown in Figure 1. All studies compared the RFA with PN in the treatment of SRTs. The baseline characteristics of the studies were summarized in Table 1.

**TABLE 1:**
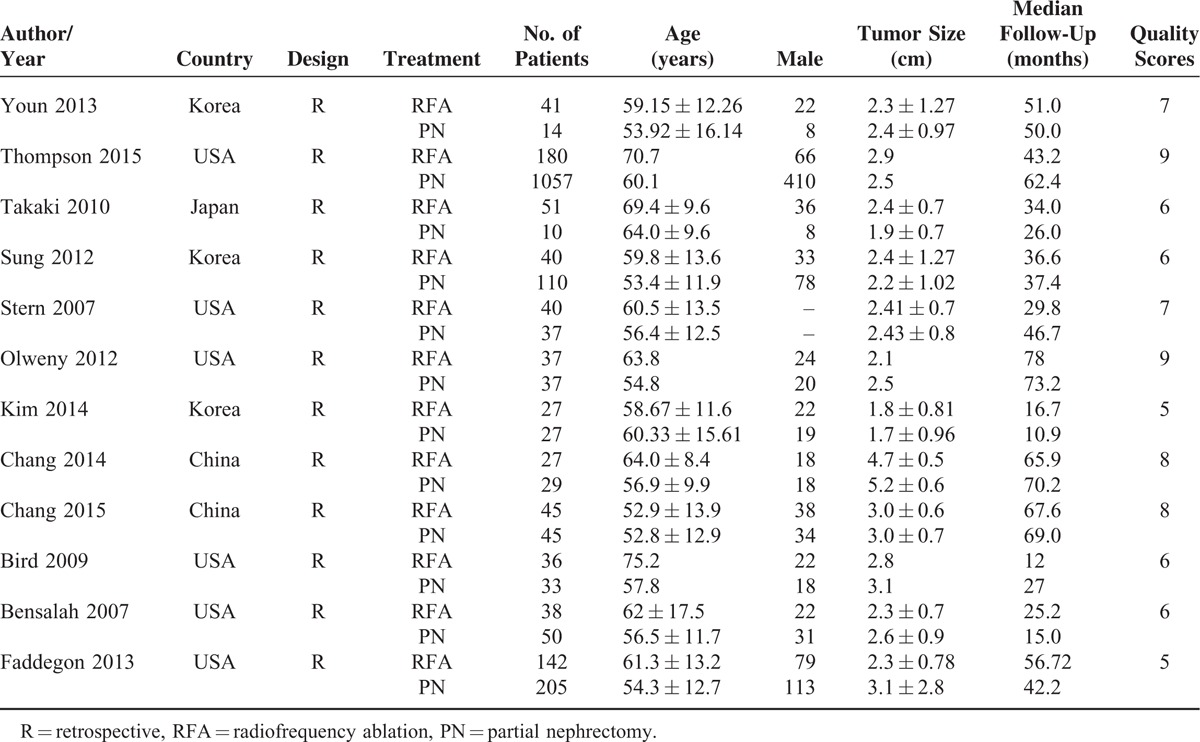
Baseline Characteristics of 14 Included Studies in the Meta-Analysis

All of the 12 studies were retrospective, nonrandomized, observational studies, and were considered to be of adequate quality for the meta-analysis according to NOS assessment (score >5 points). In total, 1654 patients treated with surgical PN and 704 patients treated with RFA were included in the quantitative data synthesis. Compared with surgical PN, patients undergoing RFA were significantly older (WMD: 6.305 years, 95% CI: 4.061, 8.548, *P* < 0.001) and had smaller tumors (WMD: −0.252, 95% CI: −0.440, −0.064; *P* = 0.008). However, there was no significant difference in gender and proportion of proven malignant tumors (Table 2).

**TABLE 2:**
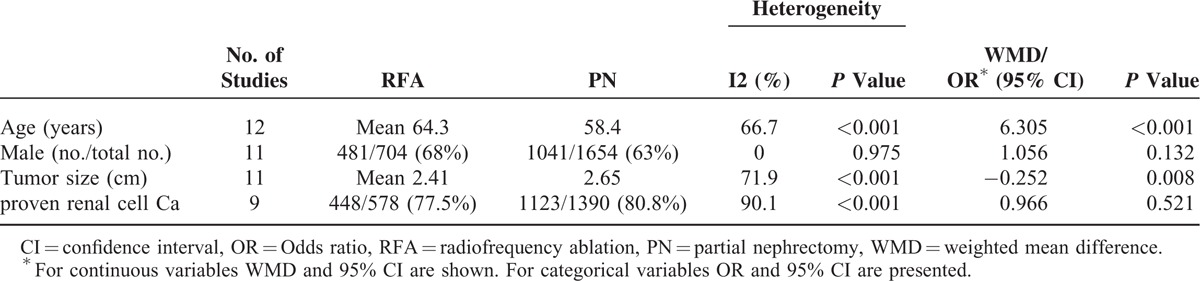
Overall Analysis of Demographic and Clinical Characteristics of Included Studies

### Perioperative Outcomes

**FIGURE 2:**
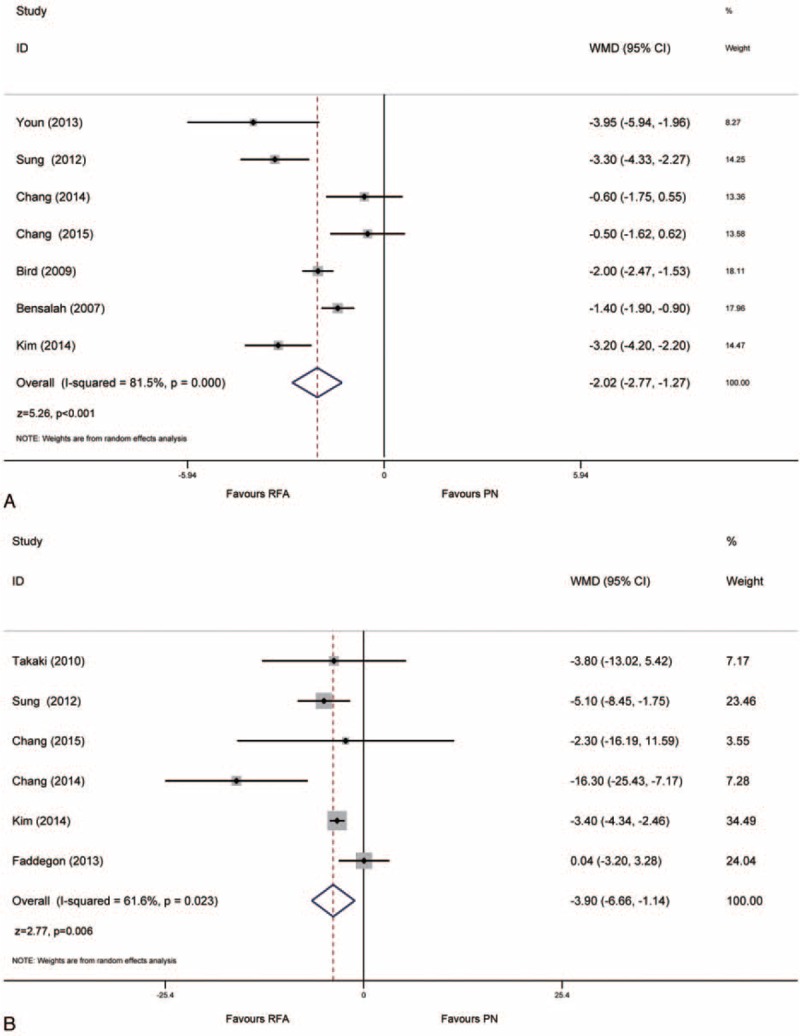
Forrest plot of perioperative outcomes after RFA and PN for LOS (A) and decline of eGFR (B). eGFR = estimated glomerular filtration rate, LOS = length of stay, PN = partial nephrectomy, RFA = radiofrequency ablation.

Length of stay (LOS) in hospital was reported in 7 studies. The patients treated with RFA had a shorter LOS (WMD: −2.02 days, 95% CI: −2.77, −1.27, *P* < 0.001; Fig. 2A). Preoperative and postoperative renal functions were also reported in 6 studies. And the pooled result indicated that RFA was associated with a significantly lower eGFR decline compared with surgical PN (WMD: −3.90, 95% CI: −6.660, −1.140, *p* = 0.006; Fig. 2B).

**FIGURE 3:**
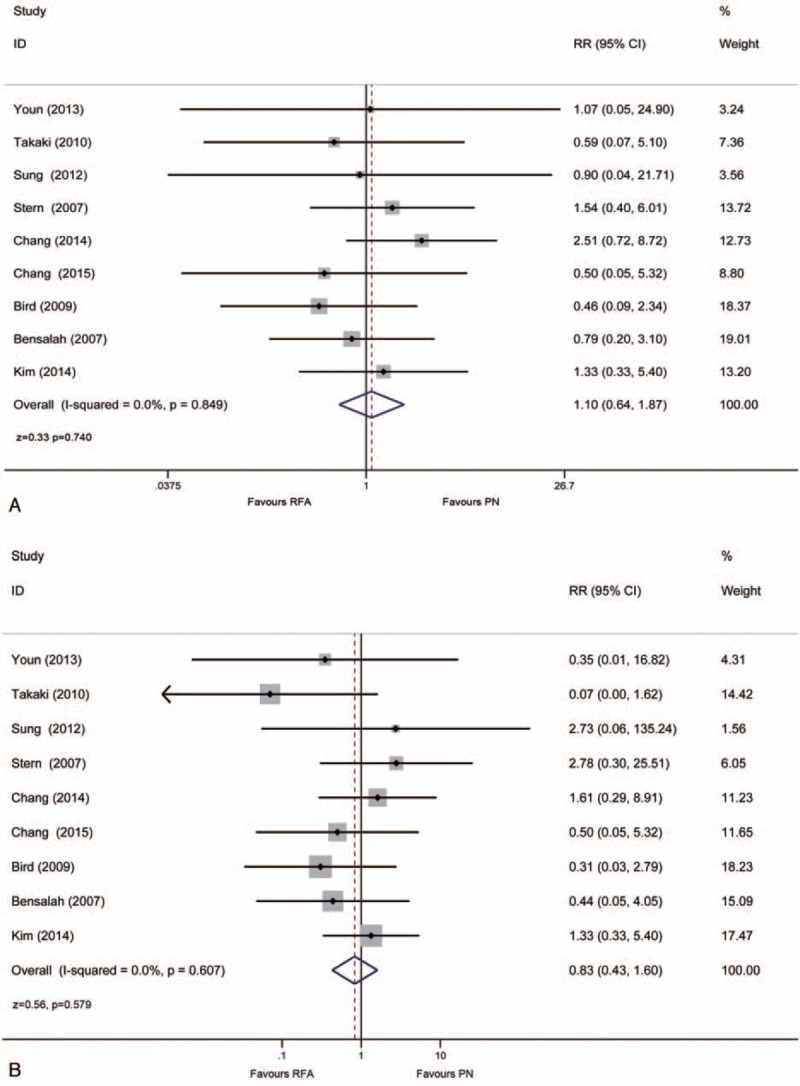
Forrest plot of perioperative outcomes after RFA and PN for overall complications (A) and major complications (B). PN = partial nephrectomy, RFA = radiofrequency ablation.

Overall perioperative complication events were described in 9 studies. The incidence of overall complications was not significantly different between the RFA group and the surgical PN group (7.5% vs 6.2%, RR:1.10, 95% CI: 0.64, 1.87, *P* = 0.740; Fig. 3A). In addition, the major complication events according to Clavien–Dindo classification could be obtained from 9 studies. The incidence of major complication was not significantly different either between the 2 groups (3.7% vs 4.4%, RR: 0.83, 95% CI: 0.43, 1.60, *P* = 0.579; Fig. 3B).

### Oncological Outcomes

**FIGURE 4:**
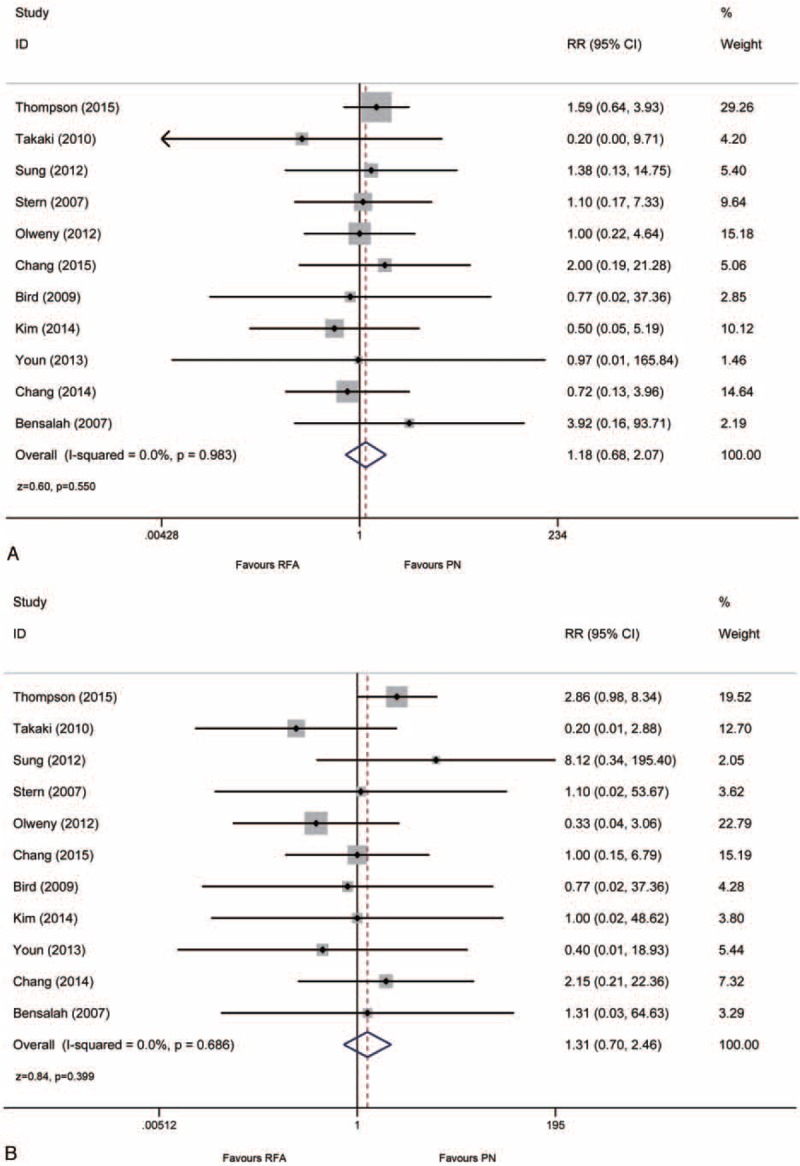
Forrest plot of oncological outcomes after RFA and PN for local recurrence (A) and distant metastases (B). PN = partial nephrectomy, RFA = radiofrequency ablation.

A total of 11 studies reported the data of local recurrence or distant metastases for patients with proven malignant disease. Eighteen cases of local tumor recurrence were reported in the RFA group versus 50 in the surgical PN group. No significant heterogeneity was indicated in these included 11 studies, and the fixed-effects model was used for the analysis. The pooled result demonstrated that the local tumor recurrence rate was not significantly different between RFA and PN groups (4.14% vs 4.10%, RR: 1.18, 95% CI: 0.68, 2.07, *P* = 0.550; Fig. 4A). In terms of distant metastases, 12 cases were reported in the RFA group and 23 in the surgical PN group. But no significant difference between the 2 modalities was indicated from the pooled result either (2.76% vs 1.89%, RR: 1.31, 95% CI: 0.70, 2.46, *P* = 0.686; Fig. 4B).

### Sensitivity Analysis and Publication Bias

To assess the stability of the results in our meta-analysis, we performed a sensitivity analysis for the analysis. The analyses regarding perioperative outcome and oncological outcome were relatively stable and credible.

**FIGURE 5:**
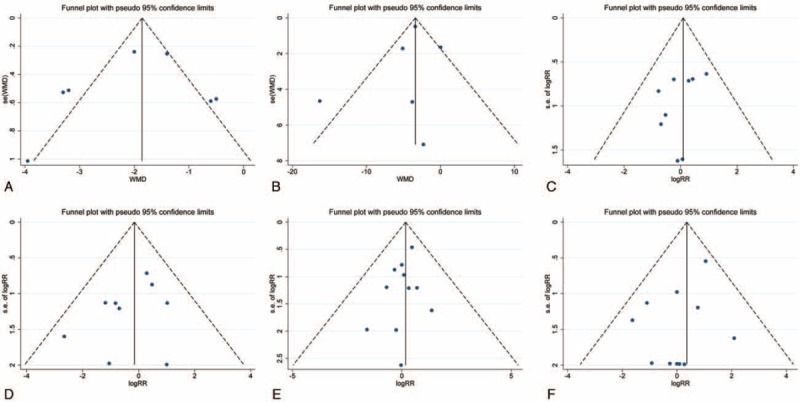
Funnel plot of publication bias for LOS (A), eGFR (B), overall complications (C), major complications (D), local recurrence (E), and distant metastases (F). eGFR = estimated glomerular filtration rate, LOS = length of stay.

We used the funnel plot, Egger test, and Begg test to evaluate the potential publication bias of the included studies in this meta-analysis. The funnel plot results did not demonstrate obvious evidence of asymmetry in all these pooled analysis (Fig. 5). Furthermore, the Egger and Begg tests did not indicate any significant results regarding publish bias in this meta-analysis (Table 3). Thus, low potential publication bias existed in our meta-analysis.

**TABLE 3:**
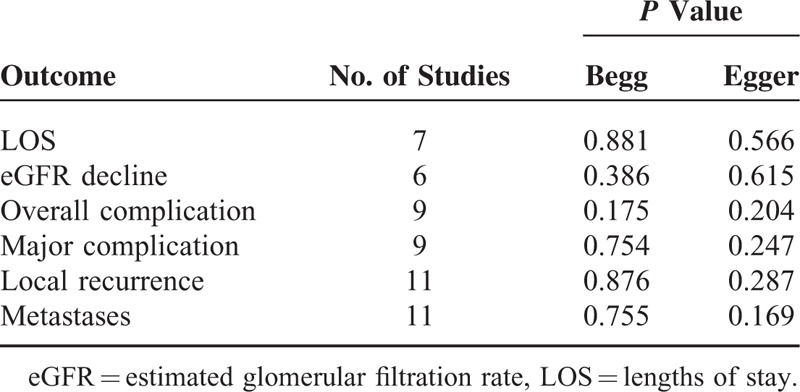
The Result of Begg and Egger Test for Publication Bias

## DISCUSSION

In the treatment of clinically localized SRTs, nephron-sparing surgery could achieve the similar oncological outcomes compared with radical nephrectomy. The estimated cancer specific survival (CSS) at 5 years were comparable between surgical PN and RN, which is 97% vs 98.5%, respectively.^31^ Surgical PN has been recommended as the standard treatment of localized RCCs.^7^

Recently, the traditional surgical modality are being challenged by the development of ablative therapies, such as RFA, cryoablation,^32^ and microwave ablation.^33^ Compared with surgical PN, RFA is associated with less operative trauma and perioperative complications. Therefore, it is more suitable for the elderly or the patients with comorbid and high ASA (American Society of Anesthesiologists) scores. Unfortunately, RCT and cohort studies of high quality that compare RFA and surgical PN directly in the treatment of SRTs are still scarce. It still remains inconsistent regarding the role of RFA for the treatment of SRTs.

Wang et al^11^ previously reported a meta-analysis comparing RFA and PN, which indicated that RFA showed a greater risk of local tumor progression compared with PN. However, the included articles were all 1-arm studies, which did not compare RFA with PN directly. So the quality of analysis and conclusion was limited. Katsano et al^34^ also performed a meta-analysis on thermal ablation, which showed that thermal ablation of small renal masses produced oncologic outcomes similar to PN. However, they pooled RFA and microwave ablation together, and did not compare RFA with PN separately. Besides, the study only included 6 available articles. In order to reduce the risk of bias, we only included the studies that compared RFA with PN directly. And after a comprehensive literature research, 12 qualified studies, which were published in the recent several years, were included in our meta-analysis. Finally, besides oncological outcomes, we also compared the perioperative outcomes including complication, LOS, decline of eGFR between RFA and surgical PN.

Oncology outcomes are the most concern for patients and doctors. In the included studies, the tumors were all localized and at low stage, and the follow-up was relatively short. So using HR of overall survival or cancer specific survival as merged variable was not appropriate. We focused on the local and distant tumor progression outcomes in this study. The pooled result showed that the differences of local recurrence rate and distant metastases rate between RFA and PN groups were not significant, which indicated that RFA may achieve similar oncological outcome for SRTs.

We also investigated the difference between RFA and PN regarding perioperative complications in our study. Nine studies reported the overall complications as well as major complications according to Clavien–Dindo classification. Our result showed that for overall complications and major complications, the differences between RFA and PN were not significant. More importantly, percutaneous RFA could be completed under local anesthesia. It is an available choice for elderly or patients with significant comorbidities that could not tolerate anesthesia or surgical trauma. And in our meta-analysis, the average age was significantly older in the RFA group than in the PN group, which reflected this advantage of RFA.

In addition, RFA was associated with shorter LOS after treatment. Compared with surgical PN, RFA could be performed percutaneously under local anesthesia. And incision and suture on renal parenchyma were not needed during RFA. So the patients could recover faster and had shorter LOS in the RFA group. Even in some studies, RFA was routinely done on an outpatient basis. So patients would be more compliant to this treatment modality.^19,29^

Furthermore, compared with surgical PN, RFA was associated with lower eGFR decline after treatment. Warm ischemia time (WIT) and amount of preserved renal parenchyma are 2 important factors that affect renal function after PN.^35^ During RFA, the renal artery does not need to be clamped, and more peritumoral normal nephrons can be preserved. Therefore, RFA causes less damage to the renal function compared with surgical PN and is more appropriate for patients with solitary kidney or chronic kidney disease.^19^

There are some limitations in our meta-analysis. First, the included studies in our meta-analysis were all retrospective observational studies. And no randomized controlled trail was identified until now. The qualities of evidence of the included studies were still poor. Second, selection bias is an inevitable problem. In the guidelines on renal cell carcinoma of EAU or NCCN, surgery is recommended as the first-line therapy for the small renal tumors (SRTs). Due to the low quality of available data, there is still no definitive conclusion regarding the morbidity and oncology outcomes of RFA. In clinical practice, most patients with smaller tumor size, younger age, and less comorbidity are treated by surgery. So a potential selection bias may exist when comparing RFA with PN in the treatment of SRTs. But it is difficult to overcome this problem through statistical methods. Prospective RCT is needed in the future. Third, in some included studies the RFA approach was percutaneous or laparoscopic, and the surgical approach was open, laparoscopic, or robotic. However, the separate outcome data of each approach were not available in most studies. Therefore multiple approaches for RFA and PN were pooled in this meta-analysis. Considering the similar efficacy on oncological outcomes,^36^ the potential bias would be low and acceptable for tumor progression. Besides, most included studies did not report the perioperative and oncological outcomes of T1a and T1b separately. The data are not applicable for this subgroup analysis, which is also a limitation of our meta-analysis. Furthermore, there was significant heterogeneity in the analysis of eGFR decline (*I*^*2*^ = 61.6%) and LOS (*I*^*2*^ = 81.5%), making the results unstable and less convincing. The renal function may be affected by some systemic diseases, such as hypertension, diabetes mellitus, and so on. The observed eGFR decline may be partially resulted from these comorbidities. But all the included studies do not report the distribution of these diseases. Finally, the definition of complications may be potentially inconsistent, such as hemorrhage. And some studies did not grade the complications according to Clavien–Dindo classification. We then graded the unclassified complications in the data extraction process if the management of complications were described. Thus the potential bias may be intensified in the analysis of perioperative complications.

## CONCLUSION

In summary, compared with surgical, RFA achieves equal oncological outcome for SRTs with similar local recurrence rate and similar distant metastases rate. And RFA is associated with similar perioperative complications, lower decline of eGFR, and shorter LOS. Therefore, RFA is an effective option in the treatment of SRTs for selected patients. Well-designed and large-scale RCTs are expected to provide more evidences of higher quality regarding the comparison between RFA and PN.
